# Sensor-based motion analysis for dementia detection: a systematic review

**DOI:** 10.3389/fdgth.2025.1698551

**Published:** 2026-01-14

**Authors:** Zongyi Jiang, Maryam Ghahramani, Nathan M. D’Cunha, Raul Fernandez Rojas

**Affiliations:** 1 Faculty of Science and Technology, University of Canberra, Canberra, ACT, Australia; 2 Biosensing & Intelligent Systems (BioSIS) Lab, Centre for Intelligent Computing and Systems, University of Canberra, Canberra, ACT, Australia; 3 Faculty of Health, University of Canberra, Canberra, ACT, Australia; 4 Centre For Ageing Research and Translation, University of Canberra, Canberra, ACT, Australia

**Keywords:** dementia, diagnosis, machine learning, motion analysis, sensor technologies, statistical analysis

## Abstract

**Introduction:**

Dementia is a progressive condition that impacts cognitive and motor functions, with early symptoms often subtle and difficult to detect. Early detection is crucial for effective intervention and improved care. Recent advances in sensor technology enable continuous monitoring of human motion, providing valuable indicators of dementia and cognitive decline.

**Methods:**

This systematic review is the first to focus exclusively on motion-based dementia detection, excluding other neurological conditions. The study aimed to address gaps in the literature by analysing evidence for motion assessment as a tool for dementia detection and by identifying and comparing sensor types, sensor placements, motion assessment tasks, extracted motion features, and analytical methods. Electronic databases (PubMed, Web of Science, IEEE Xplore and Scopus) were searched for articles published between January 2015 to May 2025.

**Results:**

A total of 23 published articles were included. Sensors used across studies included inertial measurement units, pressure mats, cameras, and passive infrared sensors, with placements on body parts, wall-mounted, or floor-based. Motion assessment tasks were grouped into three categories: gait, activities of daily living, and standing postural control. Regarding analytical approaches, 11 studies employed machine learning techniques, while 12 studies utilised statistical analysis. The findings indicate that motion-based assessments demonstrate strong potential for dementia detection, as motion-related features extracted from specific tasks can serve as sensitive indicators of dementia-related cognitive decline.

**Discussion:**

Compared with traditional dementia diagnostic pathways that often involve lengthy assessment cycles, this review's findings provide guidance on refining motion-based sensor selection, task design, and analytical methods to improve standardisation and reproducibility. Future research should prioritise: (1) large-scale, longitudinal data collection with confirmed dementia diagnoses to support machine learning model development; (2) standardisation of sensor types, placements, and motion metrics to enhance comparability; and (3) integration of multimodal data, including motion and brain signals, using explainable machine learning techniques to improve detection accuracy and clinical interpretability.

## 1 Introduction

Dementia is a progressive neurodegenerative condition that leads to a decline in cognitive abilities, memory loss, and activities of daily living (ADL) [1]. There are many types of dementia, including Alzheimer’s disease (AD), vascular dementia (VaD), dementia with Lewy bodies (DLB) and frontotemporal dementia (FTD) [2]. Dementia has become a major public health concern worldwide, especially in the context of global population ageing. Dementia affects over 55 million people globally, with nearly 10 million new cases each year. The number of people with dementia is expected to increase to 150 million cases globally by 2050 [3]. This increase is expected to place heavy pressure on healthcare systems, caregivers, and social support infrastructures, particularly in low- and middle-income countries where healthcare resources may be limited. The global cost is estimated at over USD 1.3 trillion based on the 2019 estimation by World Health Organisation, which are expected to keep rising in the coming decades [4]. Therefore, early detection of dementia is both a medical necessity and a societal imperative, given its potential to apply early intervention and slow neurodegeneration.

Currently, a diagnosis of dementia requires trained clinical staff to collect and interpret the data, along with time and effort required for hospital visits, making the process particularly challenging for older people and their caregivers. The time from initial symptom onset to a formal dementia diagnosis can take several years [5]. A diagnosis of dementia may involve a combination of methods such as reviewing medical records, interviews, questionnaires, physical examinations, cognitive assessments, laboratory testing and brain imaging [6]. In addition, early symptoms of dementia are often overlooked, leading to delayed diagnosis. Despite increased awareness of dementia, the declines in cognitive and physical functions are often seen as a normal part of ageing or are dismissed as psychological stress [7]. Developing easy-to-use, cost-effective, and accurate methods for timely detection is therefore very important.

Several studies highlight that impairments in motor function, such as altered gait patterns and postural instability, can appear in the early stages of dementia [8–10]. Recognising these motor impairments before symptoms of dementia progress could provide valuable opportunities for timely detection and intervention. To assess motor symptoms related to dementia, various gait, motion, and balance assessments have been explored. Examples of these are the postural balance test, the Timed Up and Go (TUG) test, stair climbing, and straight or back-and-forth walking tasks [11]. These assessments provide valuable insights into motor coordination and postural control. For example, compared to people without dementia, postural stability has been shown to be 32% worse in people with dementia of the same age [12]. Some studies have also shown that people with dementia face a significantly higher risk of falls [13, 14]. To detect subtle motor changes associated with dementia, appropriate sensor-based technologies, assessment tasks, and analytical methods have become increasingly important.

While various studies have explored different sensing modalities and analytical approaches, the existing literature remains fragmented in terms of sensor placement, evaluation protocols, and feature interpretation [15–17]. To gain a better understanding of recent findings in this field and identify existing limitations, a more focused review of the literature is necessary. This systematic review aims to map how sensor-based motion data have been used in dementia detection, what types of sensors and tasks have been employed, and how analytical techniques have been applied with the goal of supporting future research and guiding the development of more effective and accessible detection tools and methods.

### 1.1 Related work

Four related review articles explored sensor-based technologies and motion analysis for detecting neurodegenerative conditions. Although these reviews addressed sensor technologies in neurodegenerative research, their scopes was limited in terms of population or inclusion of different types of dementia. Boyle et al. [18] conducted an umbrella review covering sensing technologies in both Parkinson’s disease (PD) and dementia. Although PD is recognised as a risk factor for dementia, it is a different condition with different clinical manifestations and pathophysiological mechanisms. Combining PD and dementia in a single review may have overlooked the specific characteristics and needs of people with dementia. Addae et al. [19] and Dove and Astell [20] focused on technological solutions to support cognitive decline in older adults, including smart home systems and motion-based technologies. Although some of the included studies involved dementia, the reviews did not restrict inclusion to clinically diagnosed dementia populations. Their primary researches include “prevention” or “assistance” rather than focusing on detection by using motion data. Furthermore, cognitive decline (such as mild cognitive impairment, MCI) included in the scope does not necessarily progress to dementia. Puterman-Salzman et al. [21] reviewed the application of artificial intelligence for dementia detection, but their literature coverage ended in 2020, excluding more recent developments. In addition, their inclusion criteria did not specifically target clinically diagnosed dementia populations. These limitations highlight the need for a more comprehensive, up-to-date, and focused review, which this study aims to address. Table 1 shows a summary of these articles, including their scopes and limitations, with comparison to our literature review.

**Table 1:** Comparison of related reviews on motion-based dementia detection.

Author (year)	Title	# Papers	Time period	Scope & limitations (vs. our review)
Boyle et al. [18]	Activity and Behavioral Recognition Using Sensing Technology in Persons with Parkinson’s Disease or Dementia: An Umbrella Review of the Literature	9	2018–2024	Although this umbrella review includes dementia, it also covers PD, whose motor symptoms result from different neurodegenerative mechanisms than those underlying primary dementia. Furthermore, it only included nine studies, offering limited coverage.
Addae et al. [19]	Smart Solutions for Detecting, Predicting, Monitoring, and Managing Dementia in the Elderly: A Survey	64	2016–2023	This survey addresses detecting, predicting, monitoring, and assisting patients with dementia and MCI, rather than focusing specifically on dementia detection.
Puterman-Salzman et al. [21]	Artificial Intelligence for Detection of Dementia Using Motions Data: A Scoping Review	20	Inception–2020	While relevant to motion-based AI for dementia detection, this scoping review is limited to literature before 2020. Its inclusion of early symptoms or preclinical stages may result in different outcomes.
Dove et al. [20]	The Use of Motion-Based Technology for People Living With Dementia or Mild Cognitive Impairment: A Literature Review	31	Inception–2016	This review merges MCI and dementia into a single category, reducing diagnostic specificity.

These prior reviews highlight several limitations in the existing literature, including inclusion of participants without clinically diagnosed dementia, primary research focus not specifically on dementia detection using motion data, and limited temporal coverage excluding recent developments. To address these gaps, first, this review provides a synthesis of studies published over the past ten years. Second, it explores diagnostic accuracy by focusing exclusively on participants with clinically confirmed dementia. In addition, it limits the research scope to studies where motion data were used directly for dementia detection rather than for preventive or assistive purposes. Together, this provides a focused, updated, and comprehensive understanding of sensor-based motion analysis in dementia detection.

## 2 Methodology

This review followed the general principles of the Preferred Reporting Items for Systematic Reviews and Meta-Analyses (PRISMA) methodology. The PRISMA process encompasses four stages: identification, screening, eligibility assessment, and final study selection. This process was fully implemented in this review, and the study selection procedure is summarised using a PRISMA flow diagram. In addition, several PRISMA checklist items such as risk of bias assessment and certainty of evidence were not fully applicable in this review. The included studies were highly heterogeneous in sensor types, assessment tasks, extracted motion features, analytical methods, and reported outcomes. This level of variability made it infeasible to apply standardised bias assessment tools or quantitative certainty frameworks, and therefore a narrative synthesis was adopted instead. The review strategy was guided by the PICO (Population, Intervention, Comparator, and Outcomes) framework, with criteria defined as follows:

1. Population: Human participants diagnosed with dementia, along with an age-matched control group.
2. Intervention: Motion assessments conducted using sensor technologies. The motion data collected typically included parameters related to gait, posture, and balance.
3. Comparator: Motion features obtained from individuals without dementia (age-matched control group) were used as a benchmark to compare with those with dementia.
4. Outcomes: Evaluation of the effectiveness of statistical analysis, machine learning models in detecting dementia based on motion data. Specific outcomes included the identification of distinctive motion patterns associated with dementia, as well as the performance of detection models assessed through classification or regression metrics, depending on the analytical approach used.

### 2.1 Research questions

This review seeks to address the gaps in existing literature by analysing motion assessment as a tool for dementia detection. In addition, we will identify relevant sensor technologies that can be used for the objective assessment of dementia based on motion data. In this context, the main aspects are of interest: the types of motion assessments used for dementia evaluation, the types of sensors applicable for motion-based assessment, and the analytical methods employed for data analysis. Based on this scope, the research questions guiding this review are as follows:

1. What are commonly used sensor types and placements in motion-based dementia assessments?
2. What motion assessment tasks are commonly used for detecting dementia?
3. What motion-related features derived from sensor data are commonly used to detect dementia?
4. What analytical methods have been used to analyse sensor-derived motion data for dementia detection?

### 2.2 Search strategy

A keyword search was completed in PubMed, Web of Science, IEEE Xplore and Scopus in May 2025. Search terms were used in a combination, using variations of the keywords including the following : (TITLE-ABS-KEY (dementia OR alzheimer* OR lewy OR frontotemporal OR (vascular PRE/1 dementia) OR yod) AND TITLE-ABS-KEY (gait* OR motion OR movement OR motor OR walk* OR locomotion OR turning OR stepping OR balance OR postural OR sway OR stability) AND TITLE-ABS-KEY (sensor* OR (inertial PRE/1 measurement PRE/1 unit) OR imu OR accelerometer OR gyroscope OR camera OR (force PRE/1 plate) OR (pressure PRE/1 mat)) AND TITLE-ABS-KEY ((statistic* OR (machine OR deep) AND learning) OR ai OR (artificial PRE/1 intelligence)) AND NOT TITLE-ABS-KEY (protein OR chemi* OR (mild PRE/1 cognitive PRE/1 impairment*))) AND PUBYEAR > 2015 AND PUBYEAR < 2026 AND (LIMIT-TO (LANGUAGE , “English”)). In addition, the reference lists in the identified studies were examined to find additional publications of interest (i.e., snowballing).

### 2.3 Inclusion and exclusion criteria

Studies that met all of the following criteria were included in the review: (1) peer-reviewed publications in the English language; (2) studies published within the last decade (January 2015–May 2025); (3) Studies that employed at least one sensor for motion based detection, specifically using signals such as gait, balance, postural sway, or body movement patterns, obtained through walking tests, balance tasks, sit-to-stand transitions, or daily activity monitoring; (4) studies that applied statistical analysis, machine learning, deep learning, or artificial intelligence methods for dementia detection based on motion data; (5) included participants with a clinical diagnosis of dementia across any subtype of primary dementia, regardless of the time of diagnosis, as long as the diagnostic status was clearly defined.

Studies were excluded from the review if they were conference papers, letters to the editor, commentaries, or abstract-only publications; or exclusively targeted Parkinson disease dementia, as it is considered a secondary form of dementia arising from PD, rather than a primary dementia.

### 2.4 Data extraction

All search results were imported into Covidence, a web-based platform for systematic review management. Covidence was used to remove duplicate entries, screen titles and abstracts, and conduct a full-text review. Data were extracted and synthesised into tables by one reviewer (Z.J) and verified by two additional reviewers (M.G and R.F). Any disagreements were resolved through discussion. Due to the variation in study methods, participant characteristics, types of sensors used, and reported outcomes, a meta-analysis was not feasible for this review of the literature. Therefore, a narrative synthesis was conducted to identify shared insights and key patterns in the literature.

## 3 Results

In this section, the study selection process, including the steps for selecting articles in this review, is presented. Additionally, the results for each research question are provided in the following subsections.

### 3.1 Study selection

**Figure 1:**
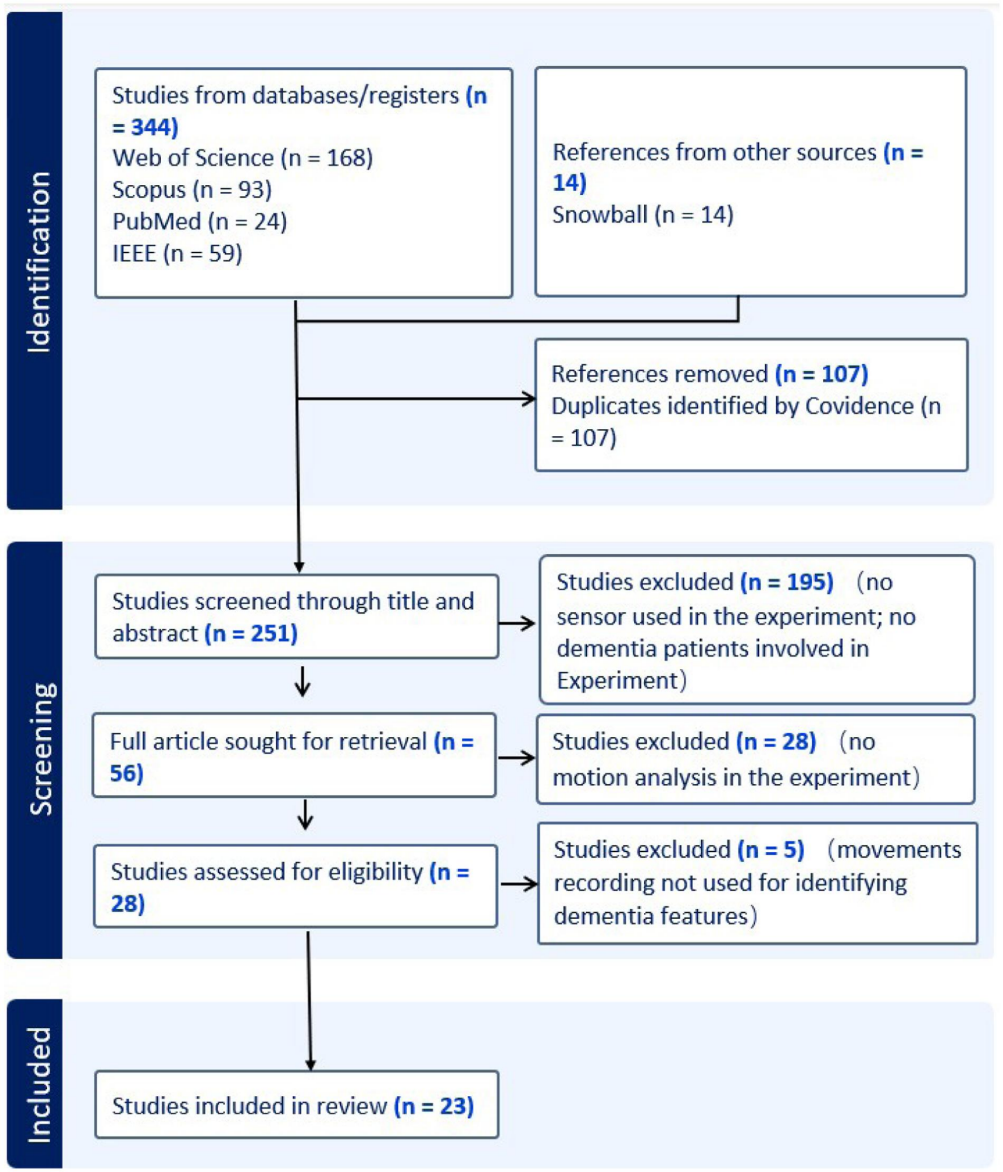
The PRISMA flow chart.

Figure 1 shows the article identification and selection process. The initial search identified 344 studies from databases/registers, distributed as follows: Web of Science (n=168), Scopus (n=93), PubMed (n=24), and IEEE Xplore (n=59). An additional 14 references were included from other sources, such as snowball sampling. After removing 107 duplicates, 251 studies remained for title and abstract screening. Subsequently, 195 studies were excluded for the following reasons: no sensor was used in the experiment, or no people with dementia were involved. A total of 56 full articles were reviewed for further evaluation. After assessing eligibility, 28 studies were excluded due to the absence of motion analysis in their experiment. Finally, five studies were excluded because, although they involved movement recordings, these data were not used for identifying dementia related features. A total of 23 studies met all inclusion criteria and were included in the review. The PRISMA flow diagram presented in Figure 1 outlines the number of records identified, screened, assessed for eligibility, and included in the final review, providing a transparent overview of the study selection process. Table 2 provides a concise summary of the included studies, highlighting key methodological elements such as participant cohorts, cognitive assessments, sensor modalities, experimental tasks, extracted features, and data analysis techniques. This information forms the foundation for the structured results presented in the following sections.

**Table 2:** Overview of reviewed studies in chronological order, analysis methodology, and type of sensor used for motion detection on people with dementia.

Author (year)	Population cohort	Cognitive assessment	Experiment	Sensor	Sensor placement	Analysis method	Extracted features
König et al. [15]	12 AD (5M/7F) (82.0 ± 8) 23 MCI(13M/10F) (77.6 ± 6.17) 14 HC (5M/9F) (74.1 ± 6.62)	AD MMSE 22.67 ± 3.6 MCI 25.5 ± 2.1 HC 28.4 ± 1.1	Walking Dual-task ADL (Lab Setting)	2D Video Camera 1080p	Wall	Statistical Analysis	– Stride length – distance travelled – Average speed – The number of missed or repeated activities
Allali et al. [16]	735 HC(374F)(73.9 ± 6.3) 394 MCI(174F)(75.5 ± 6.6) 196 Mild AD(134F)(82.5 ± 5.1) 177 Moderate AD(121F)(83.9 ± 5.6)	–	Walking (Lab Setting)	Pressure walkway	Floor	Statistical Analysis	– Stride length – Stride time – Stride velocity – Swing time – Stance time – Single support time – Double support time
Gillain et al. [17]	9 AD (5M/4F) (61.48 ± 4.85 Age) (159.05 ± 7.61H) (63.22 ± 9.35W) 4 HC (2M/2F)(60.5 ± 10.28 Age)(161.9 ± 7.41H) (60.5 ± 10.28W)	AD MMSE(26.11 ± 1.45) HC MMSE(27.25 ± 1.70)	Walking Dual-task (Lab Setting)	IMU	Lower back	Statistical Analysis	– Gait speed – Stride frequency – Stride length – Regularity – Symmetry
Fritz et al. [22]	21 DLB (13M/8F)(72.85 ± 4.58) 21 AD (13M/8F)(75.05 ± 4.96)	DLB MMSE 22.57 ± 3.57 AD MMSE 22.43 ± 4.25	TUG Shape 8 Walk (Lab Setting)	Pressure walkway	Floor	Statistical Analysis	– Velocity – Stride length – Swing time – Double support time
Yoneyama et al. [23]	13 HC 26 PD 20 AD 3 MCI 3 LBD	–	Daily Gait ADL (Real-life Setting)	IMU	Lower back	Statistical Analysis	– Cadence – Speed – Step length – Stride-to-stride variability
Costa et al. [24]	36 HC(15F/21M)(70 ± 8) 36 AD (24F/12M)(76 ± 7)	AD MOCA 11 ± 5.10 HC MOCA 25 ± 3.87	Standing Postural (Lab Setting)	IMU	Legs	SVM MLP RBN DBN	– The patient’s behaviour throughout the seven different postural tasks
Rucco et al. [25]	20 HC (12M/8F)(65.5 ± 5.4) 22 AD(12M/10F)(66.3 ± 6) 23 BvFTD (13M/10F)(66.1 ± 6.2)	HC MMSE 28.3 ± 1.0 AD MMSE 22.4 ± 4.3 BV MMSE 25.7 ± 3.0	Walking Dual-task (Real-life Setting)	3D Infrared Camera 2.8 mp	Wall	Statistical Analysis	– Speed – Stride length – Cycle time – Stride width – Swing time – Double limb support time
Nieto-Reyes et al. [26]	35 AD	–	ADL (Real-life Setting)	Smartphones with accelerometer	Pocket	Supervised learning	– Repetitive or rhythmic movements – Acceleration Characteristics – Reflecting changes in direction and smoothness of movement
Callisaya et al. [27]	89 AD 3 VaD 12 other Dementia	Mild MMSE 22.7 Moderate MMSE 16.7	Walking (Lab Setting)	Pressure walkway	Floor	Statistical Analysis	– Speed
Higuma et al. [28]	24 AD (11F/13M, 76.9 ± 4.8 Age) 9 HC (3F/6M, 74.6 ± 4.4 Age)	AD MMSE 22.5 ± 3 HC MMSE 28.8 ± 1.2	Walking (Real-life Setting)	IMU	Lower back	Statistical Analysis	– Movements of the trunk and limbs – Step-in and kick-off motion
Wang et al. [29]	62 AD (64.16 ± 4.2 Age, 160.57 ± 7.82H, 60.17 ± 8.11W) 61 HC (63.04 ± 7.70 Age, 160.19 ± 7.14H, 60.97 ± 9.9W)	AD MMSE 23.13 ± 3.76 AD CASI 79.48 ± 10.67	Walking Dual-task (Lab Setting)	IMU	Right feet Lower back	SFFS FNN	– Stride length – Number of strides – Stride frequency – Stride speed – Stride time – Stance time – Swing time
Ardle et al. [30]	17 AD (9M/8F, 67.41 ± 7.8 Age, 1.68 ± 0.09 H)	MMSE 25 ± 3	Walking (Both Lab and Real-life Setting)	IMU	Lower back	Statistical Analysis	– Step velocity – step length – step time – swing time – Stance Time
McCarthy et al. [31]	10 AD (4M/6F, 66.2 ± 5 Age, 167.9 ± 11.8H) 13 HC (7M/6F, 64.2 ± 4.1 Age, 171.2 ± 12.9H)	AD MMSE 18.6 ± 4.9	Walking straight Shape U Shape S (Lab Setting)	IMU	Shoe	Statistical Analysis	– Step time – Step length – Step hesitancy
Cheng et al. [32]	6 Dementia 8 HC	–	ADL (Real-life Setting)	PIR Sensor	Wall	Statistical Analysis SVM	– Footstep trajectory
Seifallahi et al. [33]	38 AD Female (71 ± 6 Age, 160 ± 4H, 69 ± 8W) 30 HC Female (69 ± 4 Age, 160 ± 5H, 67 ± 9W)	AD MMSE 19 ± 6 HC MMSE 29 ± 1	Walking (Lab Setting)	3D infrared paired with 2D video camera 2.07 mp	In front of the participants	SVM	– Skeleton data – Average speed – Number of steps – stride length
Ardle et al. [34]	36 AD (77 ± 6 Age, 21F, 1.66 ± 0.105 H) 45 LBD (77 ± 6 Age, 7F, 1.69 ± 0.09 H) 29 HC (74 ± 9 Age, 17 F, 1.67 ± 0.096 H)	AD MMSE 23 (14–29) LBD MMSE 24 (12–30)	Walking (Lab Setting)	Pressure walkway	Floor	Statistical Analysis	– Step velocity – Step length – Step time – Step width – Swing asymmetry – Stance asymmetry
Ardle et al. [35]	32 AD (15M/17F, 73.1 ± 6.13 Age, 1.67 ± 0.11 H, 26.03 ± 4.62W) 28 DLB (22M/6F, 76 ± 6 Age, 1.7 ± 1 H, 27.24 ± 4.82W)	AD MMSE 23 ± 4 DLB MMSE 24 ± 4	Walking (Lab Setting)	IMU Pressure Mat	Lower back floor	Statistical Analysis	– Step velocity – Step length – Step time – Swing time – Stance time
Jeon et al. [36]	13 AD (6M/7F, 73.1 ± 6.13 Age) 41 HC (16M/25F, 73.3 ± 3.53 Age)	AD MMSE 21.85 ± 5.23 HC MMSE 27.98 ± 1.64	Walking Dual-task (Lab Setting)	Pressure sole	Shoe	– DT – GBN – KNN – SVM	– Pressure – Angular velocity
Bruce et al. [37]	10 AD (8M/2F, 68.4 ± 10.82 Age) 15 HC (4M/11F, 68.4 ± 10.82 Age)	–	ADL (Real-life Setting)	3D infrared paired with 2D video camera 2.07 mp	Wall	K-fold cross-validation	– 3D position – Angular
Ghoraani et al. [38]	32 HC (23M/8F, 65.13 ± 10.53 Age) 20 AD (6M/14F, 81.4 ± 5.88 Age) 26 MCI (10M/16F, 76.81 ± 6.03 Age)	HC MOCA 26.72 ± 2.44 AD MOCA 14 ± 5.71 MCI MOCA 22.42 ± 2.33	Walking Dual-task (Lab Setting)	Pressure mat	Floor	SVM	– Stride time – Single support time – Swing time – Double support time – Stance time – Stride length
Seifallahi et al. [39]	38 AD (17M/21F, 75.75 ± 7.17 Age, 169.25 ± 9.91H, 69.25 ± 13.35W), 47 HC (22M/25F, 68.42 ± 2.60 Age, 164.71 ± 6.84H, 66.82 ± 7.78W)	AD (MMSE 23.66 ± 3.09, MoCA 19.50 ± 7.13) HC (MMSE 28.84 ± 1.06, MoCA 27.59 ± 1.87)	TUG (Lab Setting)	3D infrared paired with 2D video camera 2.07 mp	In front of the participants	SVM	– Gait features
Holloway et al. [40]	11 AD (5M/6F, 64.6 ± 5.9 Age, 168.92 ± 6.49H, 68.22 ± 13.31W) 10 Posterior Cortical Atrophy Dementia (4M/6F, 66.2 ± 5 Age, 167.91 ± 11.82H, 66.21 ± 5.03W) 14 HC (8M/6F, 64.2 ± 4.1 Age,172.36 ± 13.21H, 73.23 ± 15.23W)	AD MMSE 18.6 ± 6.1 Posterior Cortical Atrophy: MMSE 18.6 ± 5	Climbed Staircase	IMU	Heel Lower back	DT MLP	– Velocity – Stride length
Bringas et al. [41]	35 AD	–	ADL (Real-life Setting)	Smartphones with accelerometers	Pocket	A-GEM DT KNN MLP SVM	– Gait cycle variations – Acceleration rate of change

### 3.2 (RQ1): what are commonly used sensor types and placements in motion-based dementia assessments?

This section aims to identify commonly used sensor types and placements in motion-based dementia assessments, and to examine their specific applications. The studies are grouped according to sensor type and placement, highlighting the advantages, limitations, and implementation contexts of each approach.

#### 3.2.1 Sensor type

**Figure 2:**
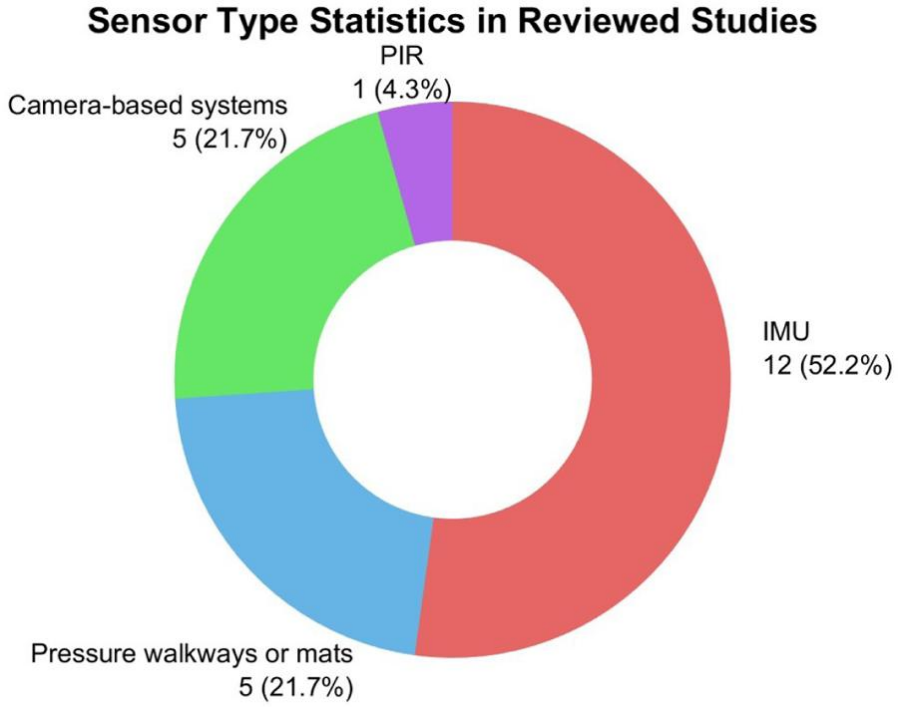
Sensor type statistics in reviewed studies.

Across 23 included studies, four primary types of sensors were employed to capture motion data in dementia-related assessments: inertial measurement unit (IMU), camera-based system, pressure walkway or mat, and Passive Infrared (PIR) sensor. Together, these sensor types highlight the diversity of technical approaches available for motion assessment in dementia research. The sensor type statistics in the reviewed studies are shown in Figure 2.

The most frequently used type was IMU, reported in 12 studies (52%), indicating its popularity in capturing human motion data in dementia-related tasks. IMU is well suited for capturing fine-grained motion features such as acceleration, angular velocity, and orientation to do gait and balance assessments. Also, the small size of IMUs allows them to be deployed in various forms to capture a broader range of body movements. For example, one study embedded IMU into wearable devices such as shoe insoles to capture foot-level dynamics [36]. Two studies used smartphone-based IMU for passive gait monitoring in daily life [26, 41]. Overall, these findings highlight the practicality of IMU, showing their advantages in motion-based dementia assessments.

Camera-based system provides a non-invasive method for capturing complex body movements, making them a valuable choice in dementia-related motion analysis. Camera-based systems were used in five of the reviewed studies (21%), demonstrating their ability in capturing body posture, joints and whole-body coordination [15, 25, 33, 37, 39] . Among these, three studies utilised the Microsoft Kinect sensor, which offers real-time 3D skeletal tracking without requiring participants to wear devices [33, 37, 39]. For instance, Seifallahi et al. [33] positioned the Kinect in front of participants during gait assessments to capture joint positions and analyse turning duration, walking velocity, and postural transitions. Another study by Rucco et al. [25] applied a motion capture system using infrared cameras mounted on the wall and reflective body markers attached to the participants’ joints. This setup allowed for measurement of joint angles and gait features, offering detailed insight into movement abnormalities. Alexandra et al. applied a video event monitoring system to not only assess gait but also track ADL, such as preparing tea or transferring between rooms, thereby providing motion data in a natural environment [15]. These camera-based systems provide a non-invasive approach to capturing comprehensive motion data for dementia detection, while enabling natural movement and imposing minimal burden on participants.

Pressure walkway and mat provide a reliable method for quantifying detailed gait parameters. Pressure walkway and mat were used in 5 studies (21%) [16, 22, 27, 34, 38]. These systems typically consist of sensor embedded walkways that detect footfalls and measure gait variables across multiple strides. For example, Ghoraani et al. [38] used a widely adopted pressure-sensitive mat to assess stride length, step width, and stance time as participants walked over the surface. Other studies leveraged similar pressure walkway to evaluate gait variability, asymmetry, and rhythm [16, 22, 27, 34]. Participants can walk without the need to wear devices, making pressure walkways and mats suitable for both clinical and lab-based environments. However, their limited size and static setup may constrain long-distance or complex mobility assessments. Despite these limitations, pressure walkways and mats provide objective data that contribute meaningfully to the evaluation of gait-related dementia analysis.

PIR sensor was the least commonly used (n=1) in dementia motion assessment in the reviewed scope. Cheng and Yang [32] deployed a network of wall-mounted PIR sensors in combination with waist-worn transmitters to detect transitional movements during ADL such as room-to-room transitions, but the gait-related parameters provided were very limited. This setup enabled the continuous tracking of mobility patterns in naturalistic home environments without relying on specific user input. Hence, PIR sensor is useful for passive behaviour monitoring in real world dementia care, particularly for detecting changes in activity levels.

#### 3.2.2 Sensor placement

Sensor placement strategies varied considerably across studies, reflecting the interaction between sensor type and assessment goals. The IMU-based studies (n=12) demonstrated the greatest diversity in sensor placement. The lower back was the most frequently targeted site (n=6), as it approximates the body’s centre of mass and enables the capture of whole-body balance and gait features. For example, Mc Ardle et al. [35] placed an IMU on the lower back to quantify postural sway and stride rhythm. Foot placements (n=4) were used to detect events such as heel-strike, toe-off, and bilateral stride symmetry. For instance, Wang et al. [29] mounted an IMU on the feet to enable analysis of stride features, such as stride length,Number of strides, stride frequency,stride speed, and stride time. Additionally, some studies (n=2) utilised smartphone embedded IMU placed in participants’ trouser pockets, allowing for passive gait monitoring during daily life without the need for specialised hardware.

In camera-based system studies (n=5), three studies positioned sensors on walls to provide a fixed field of view, while two studies placed devices in front of participants to get clear skeleton data. The PIR sensor applied study (n=1) mounted the sensors in the walls in the living house to detect transitional movements during ADL. Pressure walkway and mat (n=5) studies set the device on the floor to detect the detailed gait features.

**Figure 3:**
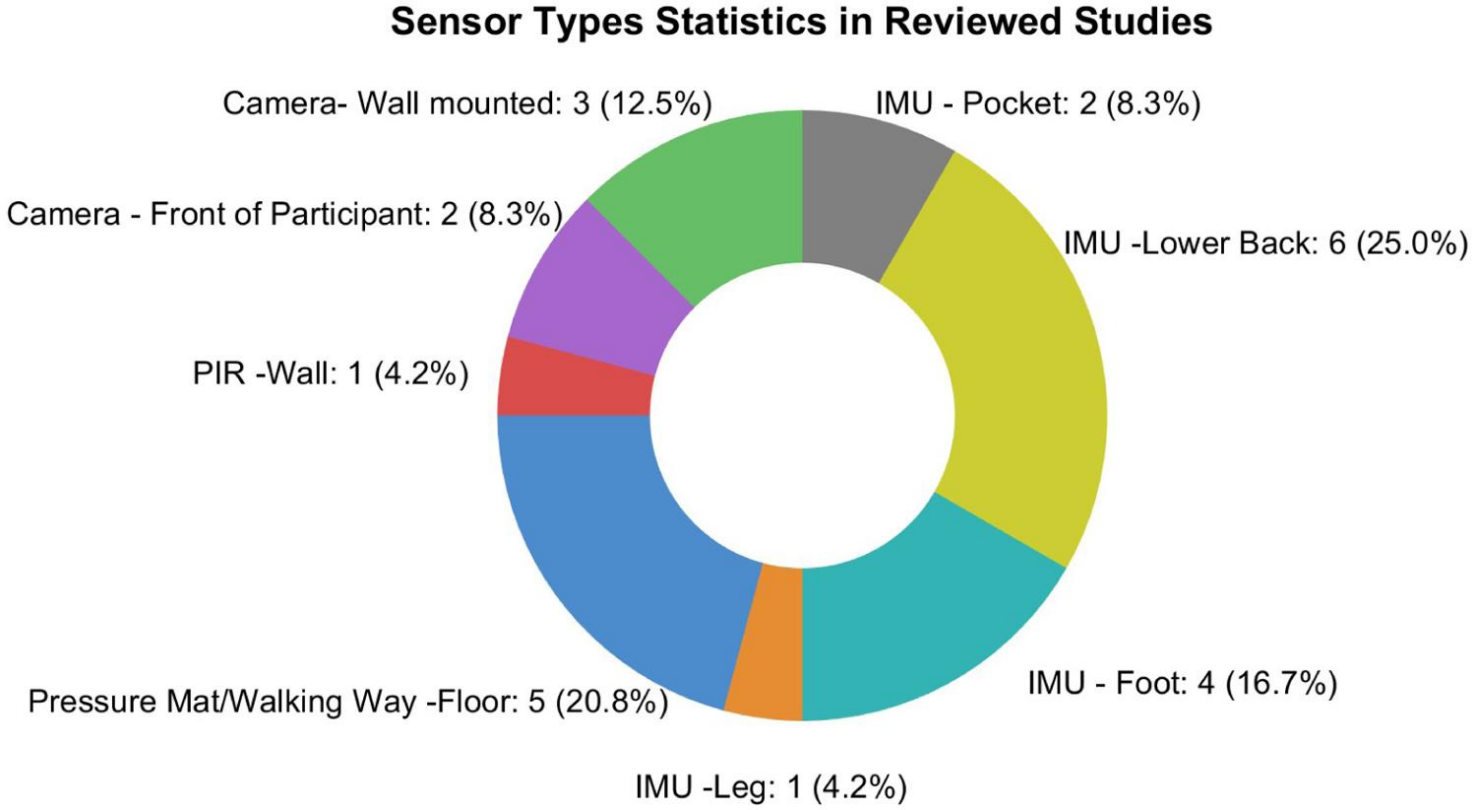
Distribution of sensor placement locations in the reviewed studies.

The sensor placement reflects a synthesis of considerations including the type of sensor used, the design of the motion assessment task, and the specific research or experimental goals. Figure 3 presents the distribution of sensor placements across the 23 reviewed studies. Each segment represents the number of studies that used a particular body location for sensor attachment, highlighting the relative percentage of placements across different body parts.

### 3.3 (RQ2): what motion assessment tasks are commonly used for detecting dementia?

**Figure 4:**
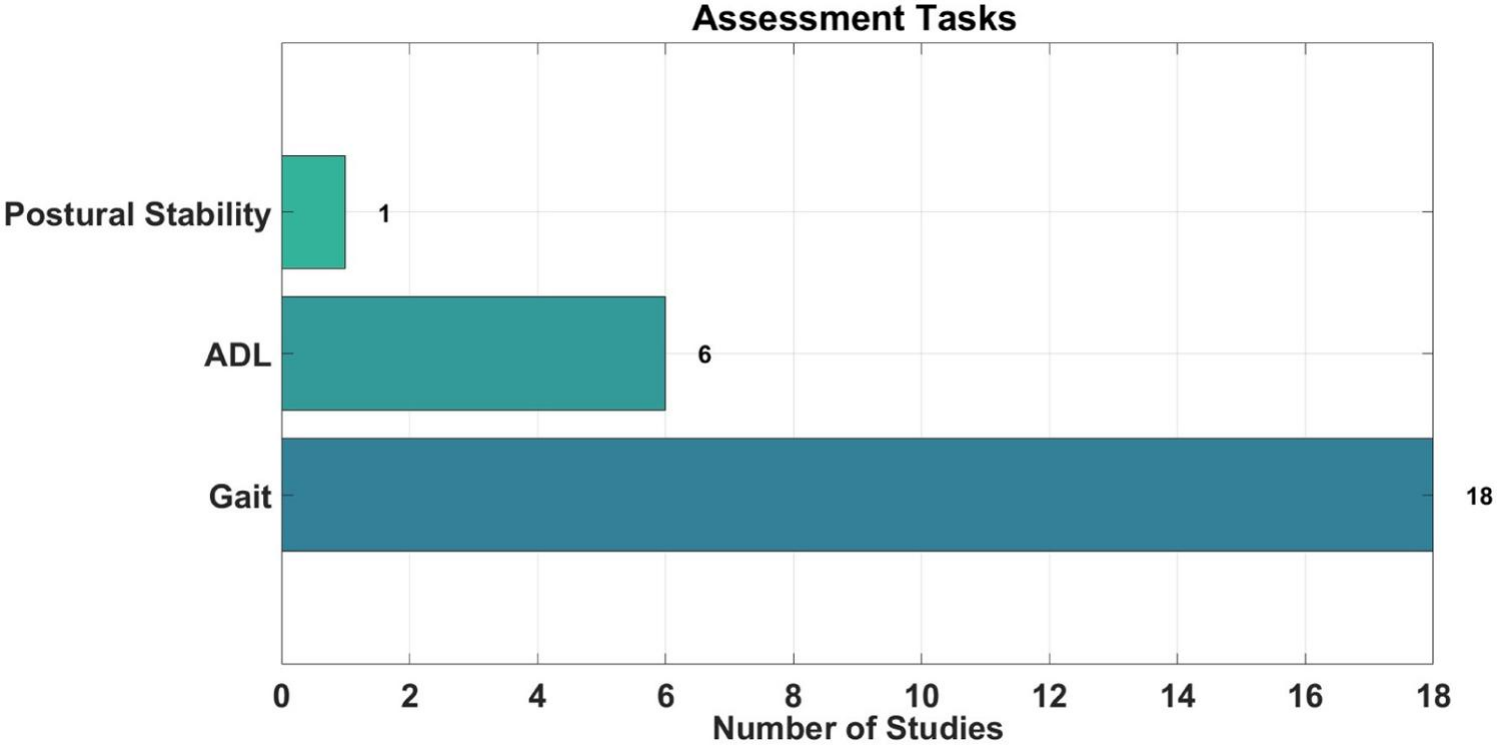
Assessment tasks statistics in reviewed studies.

Among the reviewed studies, three primary categories of motion assessments were employed to evaluate dementia-related motor symptoms: gait analysis, postural assessments, and ADL simulations. Gait assessments were by far the most prevalent, utilised in 18 out of 23 studies (78%), highlighting their role in capturing locomotor impairments. ADL-based evaluations were reported in 6 studies (26%), while postural stability assessments were observed in only 1 study (4%) shown in Figure 4. Each motion assessment type contributed a unique lens for detecting mobility related dementia symptoms across both controlled and real-life environments. The reviewed studies employed a diverse range of motion tasks to assess motor and cognitive functioning in individuals with dementia. These tasks were designed to reveal subtle changes in coordination, balance, and executive function that may not be evident through clinical observation alone. The following sections show the specific motion tasks employed under each category.

#### 3.3.1 Gait assessment tasks

**Figure 5:**
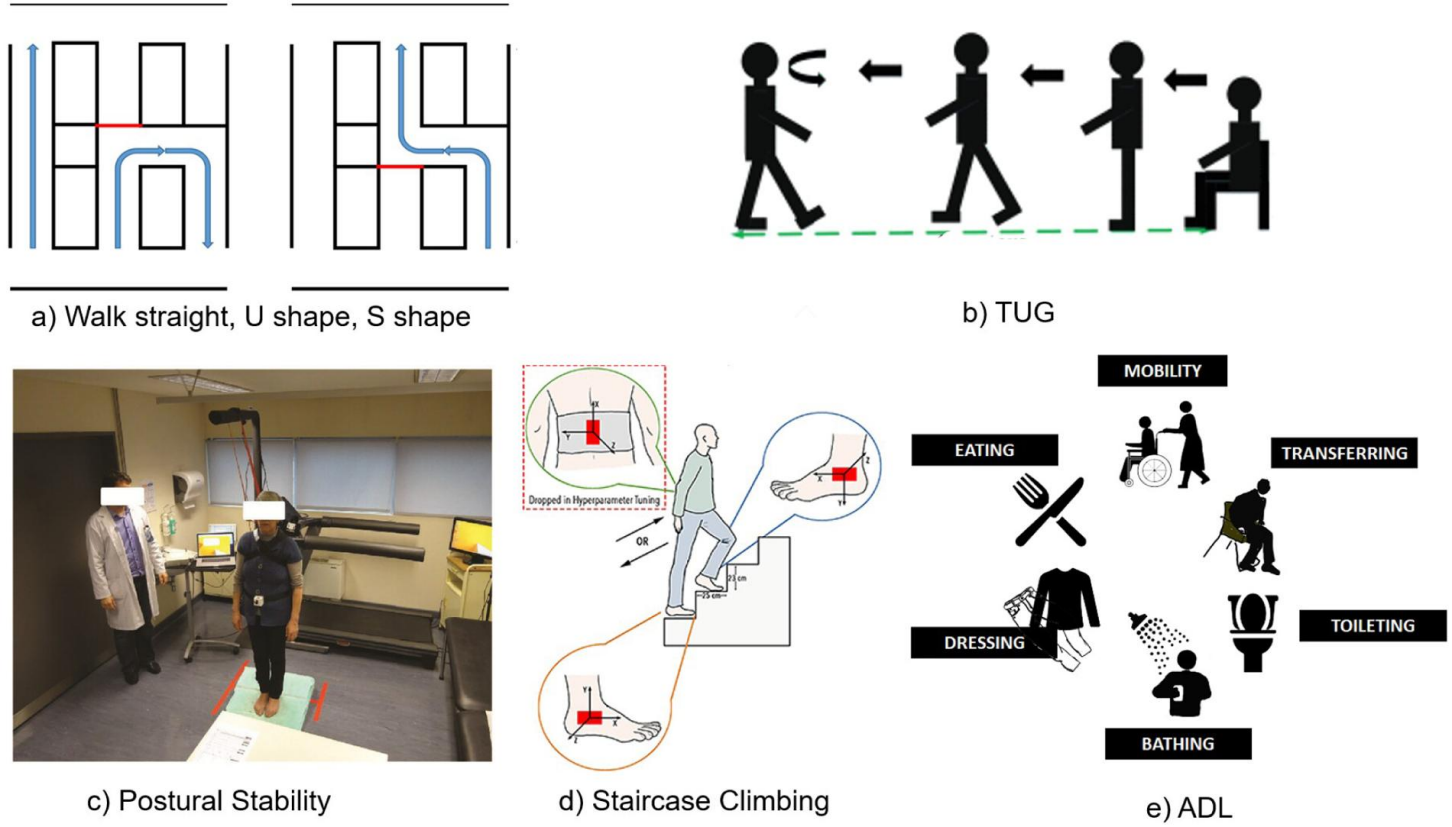
Assessment tasks commonly used for detecting dementia. (**a**) Walk straight, U shape, S shape [31], (**b**) TUG [39], (**c**) Postural Stability [24], (**d**) Staircase Climbing [40], (**e**) ADL careplanit.

Among the 18 gait assessment studies, the single gait tasks (n=12) required participants walked along a fixed path under standardised conditions. This controlled setup allows for consistent and quantitative measurement of gait parameters such as stride length, cadence, and walking speed. Besides straight walking, two studies explored more complex non-linear walking routes, shown in Figure 5a, including U-shaped, S-shaped, or figure-of-eight paths [22, 31]. These designs introduce turning and directional changes, which require higher level motor planning and balance control and these abilities are often compromised in individuals with dementia [42]. The TUG test shown in Figure 5b was used in two studies [22, 39]. By incorporating multiple phases such as sit-to-stand transitions, walking, turning, and sitting down, the TUG test effectively challenges both motor coordination and executive function. One study [40] applied a staircase climbing task shown in Figure 5d, which involves significant muscle engagement and balance control. Changes in leg lift height and forward trunk inclination during stair climbing can indicate reduced physical strength or postural stability, both of which are relevant in assessing dementia-related motor decline.

The dual-task protocols (n=6) had combined gait with a simultaneous cognitive task. The dual-task is especially valuable in dementia research because it simulates real-life scenarios where individuals walk while thinking or speaking. Since cognitive decline often begins early in dementia [43], the dual-task can reveal subtle impairments in motor-cognitive integration that may not appear during simple walking. For example, participants were asked to walk while counting backwards from 50 [17] or naming words starting with a given letter [25]. Compared to a single gait task, dual tasks introduce a higher cognitive load, often leading to observable gait irregularities, such as reduced coordination, shorter steps, or unsteady gait, which may indicate early signs of dementia. In summary, these tasks varied in complexity, ranging from a single gait task to cognitively demanding dual tasks, aiming to discover different aspects of motor function.

#### 3.3.2 Postural control assessment tasks

The Postural control task was reported in one study by Costa et al. [24], which involved static standing under altered sensory conditions, such as eyes closed or single-leg stance, this is shown in Figure 5c. This task minimised active movement and cognitive demands, allowing a clearer observation of how participants maintain balance under altered sensory conditions. Such task is particularly informative for identifying sensorimotor instability, as these highlight subtle deficits in the vestibular and proprioceptive systems. These deficits may not be captured during dynamic gait [10]. In clinical assessment, these balance-oriented tasks are especially relevant for evaluating fall risk because they help identify deficits in postural stability and sensory integration, which are key factors that contribute to balance loss and increased likelihood of falls in individuals with cognitive impairment [44]. Although underrepresented in the reviewed literature, postural assessments represent a critical dimension of motion analysis.

#### 3.3.3 ADL-based assessment tasks

Among the 23 reviewed studies, 6 studies involved ADL-based motion assessments to capture daily functional behaviours. These tasks were implemented either in smart-home simulations or through continuous monitoring in natural environments shown in Figure 5e. For example, König et al. [15] employed an automatic video monitoring system to evaluate 12 AD, 23 MCI, and 14 health control participants as they performed scripted sequences such as preparing tea, watering plants, managing medications, and writing a check. The system detected execution errors, delayed responses, or skipped steps, which are common indicators of cognitive impairment in dementia [45]. These errors reflect difficulties in planning, memory, and task sequencing, all of which are essential for independent living. It supports to facilitate behavioural comparisons between people with dementia and healthy controls. In contrast to standardised gait or postural tests, ADL-based assessments reveal real-life challenges, offering a richer context to show behavioural information, making them a potential direction for future dementia detection and intervention monitoring.

### 3.4 (RQ3): what motion-related features derived from sensor data are commonly used to detect dementia?

To quantify dementia related motor impairments, the reviewed studies extracted a diverse number of sensor-derived metrics from gait, postural control, and ADL-based tasks. These metrics were obtained using a range of sensors, including IMU, pressure-sensitive walkway or mat, PIR sensor, and camera-based system. IMU was commonly used to extract gait metrics such as stride length and cadence; pressure mats captured detailed step-by-step timing and force distribution; camera-based systems provided joint angle trajectories and posture estimations; and PIR sensor was used to collect participant’s footstep trajectory on a day. Each sensor type offers distinct insights into motor performance depending on the type of motion being assessed and the features extracted. The features are grouped into gait, postural control, and ADL to show the main findings for each motion type. Table 3 provides a comparative summary of the motion assessment tasks and corresponding Metrics used across the reviewed studies.

**Table 3:** Summary of motion assessments used for dementia evaluation.

Assessment type	No. of studies	Specific tasks	Aim of task	Sensor type	Metrics
Single-task	17	Straight-path walking (6–10 meters); U-shaped, S-shaped, or figure-of-eight paths; Staircase Climbing; TUG test involving sit-to-stand, walking, turning, and return-to-sit	To isolate and evaluate fundamental gait characteristics (e.g., rhythm, variability) under minimal cognitive load	Video event monitoring system; Pressure walkway; IMU; Kinect camera; Pressure mat	Gait speed, Stride length, Step length, Stride variability, Turn Angle, Step Count, Ascent/Descent Time, Cadence, Balance deviations
Dual-task	6	Walking while performing cognitive tasks (e.g., serial subtraction, sentence repetition, memory recall) or motor tasks (e.g., carrying a tray with water)	To assess the interaction between motor and cognitive demands, revealing impairments that may not appear under single-task walking	Video event monitoring system; IMU; Pressure mat	Gait speed, Stride length, Step length, Stride variability, Turn angle
Postural stability	1	EO, EC, EOBP, ECBP, EOFP, ECFP, and EOFS	To evaluate static balance and control under varying sensory conditions (e.g., eyes closed)	IMU	Postural Sway, Sway Velocity, Balance stability
ADL-based assessment	6	Watering plants, preparing tea/medications, reading, answering phone calls, writing checks, morning exercises in home-like environments	To measure task planning, execution, and multi-step motor cognitive function in real-world scenarios	Video event monitoring system; IMU, PIR; Kinect Camera	Task duration, sequencing errors, spatial coverage, transition frequency between activities, lingering time

Across both single-task and dual-task gait assessments, studies frequently extracted metrics such as stride length, step length, cadence, gait speed, and cycle time. These metrics are important because they reflect essential aspects of walking ability and stability, which are often disrupted in individuals with dementia. For example, Mc Ardle et al. [35] organised gait metrics into conceptual domains: pace (e.g., step velocity), rhythm (e.g., step or swing time), variability (e.g., swing time variability), and asymmetry (e.g., stance time or step length asymmetry). Among these, gait variability and asymmetry were consistently emphasised because they show strong associations with dementia-related motor cognitive impairment [46]. In addition to level walking, staircase climbing tasks have been employed to assess lower limb strength and dynamic balance during ascent and descent. These tasks are more challenging than flat-ground walking because they require greater coordination, strength, and postural control through vertical movement. Holloway et al. [40] extracted metrics such as step count, ascent or descent time, cadence, and balance deviations to evaluate motor function for people with dementia during stair negotiation. The dual-task increased cognitive load, which in turn affects gait stability and coordination, leading to changes in metrics such as step standard deviation and task duration [15]. For example, Mc Ardle et al. [30] identified step time variability and stance asymmetry as sensitive markers for detecting early stage dementia, especially under cognitively demanding conditions such as dual-task. These findings underscore the interdependence between cognitive and motor systems.

For postural control, Costa et al. [24] utilised wearable IMU placed on the trunk, thighs, and shanks to capture balance related kinematic data during seven standing tasks with varying levels of difficulties (e.g., eyes open on flat surface (EO), eyes closed on flat surface (EC), Feet together on ramp $15^{\circ }$ backward, eyes open (EOBP), feet together on ramp $15^{\circ }$ backward, eyes closed (ECBP), feet together on ramp $15^{\circ }$ forward, eyes open (EOFP), feet together on ramp $15^{\circ }$ forward, eyes closed (ECFP), and feet together on flat surface, eyes open (EOFS)). The IMU on the lower back is approximating the body’s centre of mass, which was used to estimate postural sway and body stability under different sensory conditions. It evaluated trunk angle fluctuations and estimated sway under eyes-closed or inclined surface conditions as indicators of postural control. Increased sway suggests sensory integration deficits, which are common in individuals with dementia, while minimal trunk movement reflects preserved motor coordination [47, 48].

The ADL-based tasks produced a broader and more contextualised set of features, often combining motion data with behaviour sequencing. For instance, König et al. [15] tracked activity duration, frequency, execution time, and error patterns during activities. These behavioural metrics helped identify signs of cognitive impairment, such as delays in task initiation, skipped steps, or inefficient task sequencing, which are common in early stages of dementia. Cheng and Yang [32] employed indoor positioning systems to detect step length variability and trace regularity. In another study, McCarthy et al. [31] analysed room transition frequencies and lingering time as indicators of disorientation or wandering behaviour. Irregular or disorganised movement traces may indicate spatial disorientation or executive function decline, both of which are common in early dementia [49].

### 3.5 (RQ4): what analytical methods have been used to analyse sensor-derived motion data for dementia detection?

**Figure 6:**
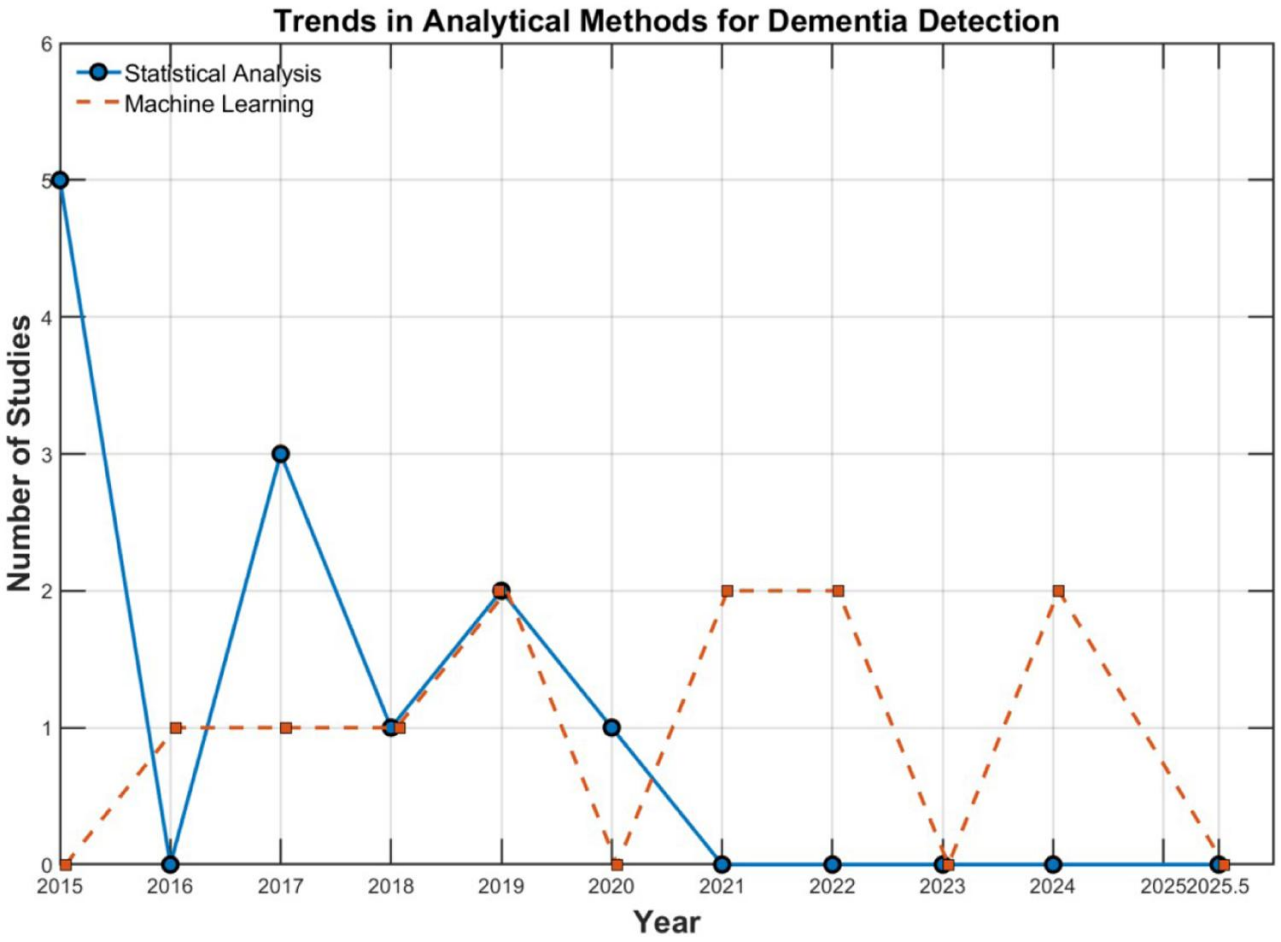
Trends in analytical methods for dementia detection.

The 23 reviewed studies employed two principal analytical frameworks to extract insights from sensor-based motion data: traditional statistical methods (n=12) and machine learning (n=11) techniques. While early studies before 2020 predominantly relied on statistical analysis, more recent research shows a clear shift toward machine learning (ML) based approaches, reflecting the growing need for data-driven dementia detection. As shown in Figure 6, the blue line representing statistical analysis methods and orange line representing ML. The statistical analysis predominated during the earlier years (2015–2019), indicating that traditional approaches were more widely used in that period. To provide a structured overview, this section is organised into two main analytical categories: (1) traditional statistical methods, which include descriptive and inferential statistics for group comparison and trend analysis; and (2) ML approaches, which focus on classification, prediction, and feature selection using sensor-derived motion data. Each subsection summarises representative studies, the specific algorithms or techniques used, and their respective applications in dementia detection.

#### 3.5.1 Statistical analysis

The traditional statistical approaches compare group means or modelling relationships using techniques like ANOVA or regression. These methods were mainly used to compare gait and motor performance variables such as stride length, gait speed, and stance time. For instance, Gillain et al. [17] applied ANOVA to evaluate gait speed and stride variability between AD and healthy controls. Meanwhile, McCarthy et al. [31] used regression analysis to investigate the predictive relationship between gait variability and cognitive scores. However, despite their interpretability, statistical approaches often assume linearity and normality, which may not hold in high-dimensional sensor data. Their reliance on small sample sizes and predefined variables may also limit their ability to capture subtle or non-linear motor changes, especially in early or atypical dementia.

#### 3.5.2 Machine learning

From 2016 onward and, particularly after 2020, more studies have begun incorporating machine learning techniques to address classification, prediction, and dementia-related motion detection tasks. As shown in Figure 6, the orange line representing ML methods rises sharply after 2020, reflecting this growing adoption. These models enabled researchers to capture complex patterns in multidimensional gait data, including both linear and non-linear relationships depending on the chosen algorithm. The most frequently applied algorithm was Support Vector Machines (SVM), featured in 7 studies, often for binary classification (e.g., AD vs. HC). For example, Seifallahi et al. [39] used SVM to classify dementia status based on gait features. In addition, Multilayer Perceptrons (MLP), Radial Basis Networks (RBN), and Deep Belief Networks (DBN) were employed to model more complex and non-linear associations. Simpler classifiers like k-Nearest Neighbors (KNN) and Gaussian Naive Bayes (GNB) were used primarily for comparison or benchmarking purposes [36]. To enhance robustness and performance, some studies adopted ensemble learning methods, including Decision Trees (DT), Random Forests (RF), and Gradient Boosting algorithms. These were used both for classification and feature importance ranking. For example, Mc Ardle et al. [34] applied DT, GNB, SVM, and KNN to classify participants based on gait asymmetry during dual-task. Furthermore, many studies used cross-validation methods like K-fold to make sure their models worked well on new data. Despite their analytical power, many ML models are treated as black boxes, which raises concerns about clinical interpretability and decision transparency. While traditional statistical approaches remain essential for initial group comparisons and interpretability, machine learning methods are increasingly used to support predictions and explore potential non-linear patterns in motor behaviour. Together, these methods provide complementary strengths for advancing sensor-based dementia assessment, as summarised in Table 4.

**Table 4:** Summary of statistical and machine learning methods used for dementia detection.

Category	Method/model	Analysis description	Evaluation metrics	Task type
Statistical analysis	ANOVA, Regression, t-test	Group comparison, correlation modelling	p-value, variability coefficient	Classification/Regression
	Skewness, Kurtosis	Normality testing	–	–
	Naïve Bayes (simple)	Probabilistic modelling using Bayes theorem	Accuracy, Cross-validation	Classification
	Space-Time Tracking	Movement comparison algorithms	Variability measures	Classification
Machine learning	SVM	Linear/nonlinear classification, kernel optimization	Accuracy, F1-score, AUC	Binary/Multiclass
	Decision Trees (RF, GB, XGB, AdaBoost)	Feature importance, ensemble learning	Precision, Recall, AUC	Classification
	MLP, DBN, RBN, FNN	Neural modelling with dense layers, feature selection (SFFS)	Accuracy, RMSE, $R^{2}$	Classification/Regression
	KNN, GNB, GP	Distance/probabilistic models, graph modelling	Accuracy, AUC	Classification

## 4 Discussion

In this review, we synthesised findings from 23 published studies that employed sensor-based motion assessments in individuals with dementia (Table 2). Our findings indicate that IMU is the most commonly selected sensor for capturing dementia-related motion data due to its cost-effectiveness, portability, compact size, and ability to record multidimensional motion. It allows IMU to be attached to various parts of the body or embedded in shoes and smartphones, making it suitable for long-term monitoring of dementia-related motion in real-world settings. Regarding sensor placement, we found that the lower back was the most frequently targeted location for dementia motion assessment. This region closely approximates the body’s centre of mass, making it an optimal location for capturing whole-body balance and gait features. In terms of motion-based dementia assessment tasks, straight walking was the most widely used task, where participants walked along a predefined path under standardised conditions. Such controlled environments enable consistent and quantitative extraction of gait parameters.

Other findings are about extracted features and analytical methods for dementia detection. The frequently extracted features include stride length, step length, cadence, gait speed, and cycle time. These features are important because they reflect essential aspects of walking ability and stability, which are commonly affected in people with dementia. The analytical methods used to process these extracted features have shown changes over the timeline. Earlier studies (pre-2020) primarily relied on statistical analyses, using descriptive and inferential techniques for group comparisons and trend identification. In contrast, more recent studies demonstrate a clear shift towards machine learning approaches focused on classification, prediction, and feature selection. Among machine learning models, SVM was the most frequently applied model.

There are distinctly different aspects from prior reviews. First, we exclusively included studies involving clinically diagnosed dementia and intentionally excluded other neurological conditions that may cause motor impairments but are not necessarily associated with dementia. This ensures that the motion features summarised in this review are directly relevant to dementia detection. Second, we systematically examined sensor placement strategies and deployment configurations to understand the most effective locations for motion capture, the experimental requirements of different sensor technologies, and the potential intrusiveness for people with dementia. In addition, this paper reviewed the motion features that can be extracted for dementia detection. This is essential for discovering effective biomarkers of dementia-related motor changes and provides a valuable foundation for selecting appropriate analytical methods. To deepen the interpretation of these findings, we structure the discussion around our four research questions, highlighting the advantages, limitations, and practical implications of each component of the reviewed studies.

### 4.1 Sensor technologies in dementia motion assessment

The reviewed articles covered four types of sensor technologies: IMU, pressure walkway or mat, camera-based system, and PIR sensor, aimed at detecting dementia-related motion data. IMU is the most commonly used sensor in dementia detection researches due to their cost-effectiveness, mobility, compact size, and ability to capture multidimensional motion data. Its compact size makes it adaptable to different experimental tasks. For example, IMU can be attached to specific body parts (e.g., pelvis or heel) to capture motion data during stair-climbing activities [40], whose scenario can hardly be replicated through pressure walkways or mats. However, as a wearable sensor, it is important to consider that people with dementia, especially older people, may experience discomfort, resistance, or stress when asked to wear such devices. This resistance may arise from unfamiliarity with the devices or from physical discomfort. A systematic review of the experiences of older people with wearable devices highlighted that they are more likely to accept devices that are easy to attach, secure, unobtrusive to clothes, and simple to handle [50]. From our reviewed articles, some studies had attempted to embed IMU into familiar objects (e.g., shoes, phones) [36, 41], which could also be suitable for long-term monitoring in real-world settings, though such applications remain underexplored in dementia.

The camera-based system, as a non-wearable technology, provides highly accurate motion data for people with dementia during assessments without requiring body-mounted devices [33, 37, 39]. This system can provide markerless tracking of skeletal joints and body segments, providing rich spatial and temporal data for analysing gait, balance, and postural transitions [37]. The reviewed studies employed both 2D video cameras and 3D depth sensors, with the latter capturing skeleton coordinates [33, 39]. In terms of adaptability, camera-based systems are well suited for tasks performed within a constrained field of view such as straight-line walking, turning, or sit-to-stand transitions, but they become less flexible for long-path or multi-room assessments because participants must remain within the camera’s visible range [33]. Their deployment typically requires controlled lighting and fixed positioning in laboratory or clinical settings [51], which may be challenging for older people with dementia, as conducting movement assessments in participants’ living environments may be safer and easier than asking them to travel to a lab.

In addition to IMU and camera-based system, other sensor technologies such as pressure walkway/mat and PIR sensors have been employed to assess motion in people with dementia [32, 52], each serving distinct purposes depending on the context. Pressure walkways and mats are particularly used in controlled laboratory environments to capture high precision spatio-temporal gait parameters such as step length, stride width, cadence, and stance or swing time. Their use is restricted by their limited physical coverage [52]. The application of PIR sensors appeared in only one of the reviewed studies [32]. PIR sensors provide monitoring of activity patterns and behavioural transitions in home or care settings, but their gait-related parameters are insufficient to serve as the sole source of information for dementia detection [32]. Nevertheless, they have potential for supporting long-term behavioural tracking of people with dementia in real-world contexts. The choice of sensor type should be based on the aims and objectives of each study, as well as the advantages and limitations of the respective sensors. The choice of sensor type should be based on the aims and objectives of each study and the advantages and limitations of each sensor, and incorporating sensor fusion may offer a promising direction for future research. For example, researchers can integrate IMUs with 3D depth cameras to combine wearable high-frequency kinematics with precise spatial tracking [53].

### 4.2 Motion assessment tasks

Motion assessment tasks, including single-task, dual-task, postural stability, and ADL, provide different advantages for motor function evaluating. The single gait task is widely adopted due to their simplicity, standardisation, and reproducibility. It allows for consistent data collection across different environments and groups. For instance, the TUG test uniquely integrates multiple movement phases (sit-to-stand, walking, turning, and stand-to-sit), making it well suited for capturing transitional dynamics and postural adjustments [39]. Complex walking paths like figure-of-eight and S-shapes are particularly valuable for assessing spatial navigation and turning ability, because they impose navigational demands that resemble real-world challenges and therefore may capture early deficits in executive control and visuospatial planning [22]. The staircase climbing task further extends assessment to vertical mobility, motor planning, limb coordination, and dynamic balance. Its vertical complexity adds an additional challenge beyond flat ground walking, requiring anticipatory postural adjustments and limb timing that may uncover deficits not seen in other gait tasks [40]. However, the single gait task is limited to a predefined and fixed setting, and therefore cannot capture broader mobility in real-world environments.

While retaining the standardisation and reproducibility of single-task protocols, the dual-task more closely mirror real-world mobility challenges by engaging additional cognitive or motor requirement. It better reflects everyday mobility, as individuals rarely walk without cognitive or motor distractions. The findings from dual-task reviewed studies consistently underscore the heightened sensitivity of dual-task to subtle impairments that may not be observable under single-task conditions. Moreover, different types of additional cognitive or motor load can show which neural systems are stressed, and thereby reveal specific deficits. For example, serial subtraction tasks test working memory and attention, while verbal fluency tasks assess executive function and word retrieval. Motor tasks, like carrying objects, challenge planning and multitasking [54–56]. This diversity highlights the need for careful task design. Future work may consider dual-task designs aligned with specific dementia subtypes and severity levels. Hence, dual-task assessments should not be seen as a mere extension of gait analysis, but as a flexible and scalable approach that may substantially enhance early-stage dementia detection.

Postural stability and ADL-based tasks each address distinct aspects of motor control and functional capacity in dementia assessment. The postural stability task was included in only one study, which was used to assess balance and equilibrium maintenance under altered sensory conditions [24]. Rather than simply measuring sway or stance duration, this task revealed how people with dementia adapt to changes in sensory input, reflecting impairments in sensorimotor integration and cognitive control mechanisms that are often disrupted in dementia. The ADL-based tasks simulate real-world activities. These tasks are designed to resemble real-life activities, requiring participants to plan actions, switch between tasks, and correct mistakes, providing a more realistic assessment of executive functioning [26]. Overall, postural stability and ADL-based tasks can serve as valuable complements in motion-based dementia analysis; however, when used alone, the motion data they provide may be insufficient for reliable dementia detection.

These diverse motion assessment tasks provide a multi-layered lens into dementia-related motor and behavioural changes. Future work could consider to move toward integrative designs that combine different task types into unified protocols, thereby capturing gait, balance, and functional behaviour simultaneously. For example, Fritz et al. [22] integrated both the TUG and figure-of-eight walking tasks using wearable sensors, demonstrating how combining structured, phase-based assessments with complex navigation challenges can capture a broader spectrum of motor behaviours. The different task types integration approach enhance sensitivity to subtle mobility impairments that might be overlooked when using a single type of task.

### 4.3 Motion related features

Different motion-related features were extracted from specific assessment tasks, which can serve as sensitive indicators of cognitive decline. For instance, reduced speed and increased step variability from gait assessments are consistently associated with global cognitive impairment and elevated fall risk. These associations are thought to reflect compromised neural control of locomotion, particularly due to disruptions in attention, motor planning, and sensorimotor integration [57]. The complex paths and turning tasks add more valuable features for analysis, such as turn duration, hesitation before directional change, reduced angular velocity, and erratic path trajectories. Such features highlight the intricate interplay between motor planning, visuospatial processing, and cognitive load [22]. The TUG test also enables extraction of phase-specific features such as sit-to-stand duration, peak acceleration during turning, and time to stabilise after sitting down [39]. These transitions are cognitively demanding, as they require anticipatory postural control, making them particularly sensitive to subtle deficits in executive function and attention. Moreover, performing a cognitive task while walking usually reduces walking speed, increases step-to-step variability and gait asymmetry, and lengthens double-support time [58]. These dual-task effects can amplify subtle gait changes and therefore make such features easier for comparing individuals with dementia to healthy controls, supporting dementia detection [59].

The motion metrics derived from postural task and daily activities also can provide useful information about motion changes in dementia. The postural task typically provides features like centre of pressure sway, sway velocity, and postural variability, which are highly sensitive to deficits in sensory integration and attention regulation [24]. Hence, combining gait features from TUG with balance features from postural tasks can provide a more comprehensive motion analysis for dementia detection. For ADL-based assessments, extracted features provide insights into general behavioural changes including reduced mobility or irregular daily routines. These features changes can help to evaluate cognitive or physical decline in dementia [32]. The strength of ADL features lies in their potential to enable large-scale data collection over weeks or months, allowing for the early detection of behavioural drift, habit changes, and increasing task fragmentation.

### 4.4 Analysis methods: statistical and machine learning approaches

Traditional statistical methods were used primarily to evaluate group differences and test predefined hypotheses, offering interpretable insights from relatively small datasets. While methods such as ANOVA and regression can effectively capture average group-level differences, they often fall short in revealing the subtle, non-linear, and multivariate patterns that are crucial for understanding dementia related motor decline. This limitation is especially relevant for high-dimensional, time-series sensor data analysis. Furthermore, many traditional methods tend to rely on assumptions of normality and independence that are often not fully met in ecological motion data, and their dependence on group-level averages can make it difficult to account for individual level variability. Although traditional statistical approaches remain valuable for establishing population-level baselines and testing predefined hypotheses, they are increasingly limited in their ability to model the complex, heterogeneous, and evolving nature of motor behaviour associated with dementia. These limitations have driven a methodological shift toward machine learning techniques.

Machine learning techniques have been increasingly used in recent dementia motion studies to classify individuals, identify important features, and detect complex patterns in high-dimensional sensor data. Researchers are increasingly using more advanced model architectures such as MLP, RBN, and DBN to better model non-linear and multi-class problems [40, 41]. However, while these models improve sensitivity to complex patterns, they also face challenges including overfitting on datasets, which may hinder clinical adoption [60]. In the future, ensemble methods such as gradient boosting (e.g., XGBoost) could be considered, as they offer strong predictive performance and can help mitigate overfitting through model aggregation and regularisation [36].

Across the studies that employed machine learning models, the reported predictive performance varied widely depending on the sensor modality, feature set, and study design. Most classification models achieved accuracy values ranging from approximately 75% to 92% [24, 33], with SVM being the most frequently used algorithm. Models incorporating spatiotemporal gait features, particularly gait speed, step variability, turn duration, and dual-task gait parameters, tended to achieve the highest discriminative performance between dementia and healthy controls [39]. However, only a minority of studies reported comprehensive performance metrics such as F1-score or accuracy, limiting cross-study comparability [38, 39].

Machine learning also has the advantage of handling longitudinal or multi-session datasets, which are important in dementia motion studies because motor and cognitive abilities change gradually over time. For example, one study has implemented continual learning frameworks [41], which allow models to incrementally learn from new data without forgetting previously acquired knowledge. By enabling adaptation over time, continual learning improves the ability to track progressive changes in motor and cognitive function, potentially enhancing both personalised assessment and early detection. However, the relatively limited sample sizes of the included studies, as shown in the Population Cohort column of Table 2, constrain robust model training, and the scarcity of independent validation datasets hinders the translation of findings into clinical practice [40]. Future work should involve multi-centre collaboration to establish shared large-scale databases, supporting both machine learning model training and independent validation.

### 4.5 Limitations and future work

Despite the comprehensive scope and systematic methodology, this review has several limitations. First, the reliance on four widely used academic databases (Web of Science, Scopus, PubMed and IEEE Xplore) may have limited the range of studies included, meaning that there is a possibility that some relevant studies were missed. Second, the review was limited to English-language publications indexed in selected databases, which may have led to the exclusion of important research published in other languages or less accessible sources. Third, the reviewed studies included participants with an uneven sex distribution, which may affect the generalizability of the findings. In addition, although participants in the reviewed studies were screened for cognitive and basic motor function, coexisting conditions affecting motion could not be fully and easily excluded. Hence, most studies employing statistical or machine learning approaches have focused primarily on dementia effects without accounting for multi-morbidity.

The findings and limitations in this review point to several directions for future work. First, future reviews may benefit from broader inclusion criteria and multilingual database coverage to ensure a more exhaustive synthesis of the field. Second, future studies should aim to recruit a more balanced sample in terms of sex to improve the generalizability of findings. Third, to better focus on the effects of dementia on motion, future studies could include simple screening of common coexisting conditions and control for their potential influence when analysing motion data. Furthermore, current research on outside-of-lab approaches is insufficient. Future work could consider refining the design of such approaches to address this gap.

## 5 Conclusion

In conclusion, this systematic review highlights the current progress in sensor-based motion assessment for dementia detection. From a theoretical perspective, it provides evidence that specific motion features are closely linked to dementia-related changes, supporting their value as objective biomarkers. From a practical standpoint, the findings indicate that IMU sensors are the most widely adopted due to their affordability, portability, and ability to capture multidimensional motion data. Dual-task assessments have shown particular promise, as they better reflect real-world mobility demands by integrating cognitive and motor components. In addition, ML has advanced dementia motion analysis by modelling complex and high-dimensional data better than traditional methods. Based on these findings, future work could consider further refine the design of outside-of-lab approaches on motion-based dementia detection to address current shortage in this research area. Future studies should also consider screen common coexisting health conditions and account for their effects on motion data. Such efforts would directly enhance the reliability and effectiveness of motion-based dementia detection in practice.

## Data Availability

The raw data supporting the conclusions of this article will be made available by the authors, without undue reservation.
